# Prevalence of broncopulmonary and otorhinolaryngologic symptoms in children under investigation for gastroesophageal reflux disease: retrospective analysis

**DOI:** 10.1590/S1808-86942011000300010

**Published:** 2015-10-19

**Authors:** Victor José Barbosa Santos, Giovana Tuccille Comes, Tatiana Maria Gonçalves, Mary de Assis Carvalho, Silke Anna Thereza Weber

**Affiliations:** 1 ENT; Graduate Student - Basis of Surgery - FMB-UNESP; 2 Medical Student; 3 3rd Year Resident in ENT - FMB-UNESP; 4 MSc; Professor; Attending Physician - Pediatric Gastroenterology Program - Department of Pediatrics of the Medical School of Botucatu - Unesp; 5 PhD in Basis of Surgery - FMB-UNESP, Professor of Otorhinolaryngology - Medical School of Botucatu -Unesp

**Keywords:** child, gastroesophageal reflux, respiratory signs and symptoms

## Abstract

Gastroesophageal reflux disease (GERD) is a common ailment in children, adding up to the evidence that gastroesophageal reflux is an important cofactor in upper airway disorders, especially in the pediatric population. It is very common for it to impact the upper and lower airways. Our goal was to assess the presence of otorhinolaryngological symptoms in children aged between one and twelve years in whom gastroesophageal reflux is suspected.

**Materials and methods:**

We assessed data from the charts of patients up to 12 years of age submitted to 24 hour pH measuring of one of two channels, placed at 2 and 5 cm from the LEE in order to confirm the diagnosis of Gastroesophageal Reflux Disease.

**Results:**

We studied 143 charts from children who underwent 24 hour pH measuring to investigate GERD; however, only 65 were included. The most prevalent symptoms in the children were bronchopulmonary, found in 89.2%, of sinonasal symptoms (72.3%), otologic (46.1%) and repetition UAW infections (44.6%). When we compared the presence of each group of symptoms of the results from the pH measuring, no significant differences were found between the symptoms and the pH measuring results.

**Conclusion:**

GERD can manifest in different ways and otorhinolaryngological symptoms are frequent in children.

## INTRODUCTION

The Gastroesophageal Reflux Disease (GERD) is a common disorder of childhood and, each year we have more evidence that the gastroesophageal reflux is an important cofactor contributing to airway disorders, especially in the pediatric population. The Brazilian Consensus in Gastroesophageal Reflux Disorder (CBDRGE) defined GERD as a chronic disease arising from the retrograde flow of the gastro-duodenal content to the aesophagus and/or adjacent organs, causing a variable spectrum of esophageal and extraesophageal signs and symptoms, with or without tissue lesions^1^.

The gastric content reflux is a physiological occurrence which happens more frequently to infants, especially premature babies, and it reduces as they grow older, disappearing around 24 months of age. In the presence of pathological conditions, however, either by the increased frequency of reflux episodes, or by the longer time of exposure of the gastric mucosa to the acid, there are clinical and histopathological consequences known as Gastroesophageal Reflux disease^2^.

The Lower Esophageal Sphincter (LES) in the distal end of the esophagus has attracted the interest of those trying to explain why infants have a greater predisposition to develop GERD when compared to older children and adults. However, animal models and human studies, have suggested that the LES muscle is not weaker than in the adult, nonetheless, there is an inadequate relaxation of this muscle, since its maturing will only happen at around 3 months of age, when the LES which was intra-thoracic, starts having an intra-abdominal portion^3^.

It is very common, especially in children, besides the classic pyrosis, seeing manifestations in the upper and lower airways^4^, ^5^, ^6^, ^7^, ^8^, ^9^. Among them, we can mention recurrent tonsillitis, recurrent pharyngotonsillitis, asthma, recurrent otitis media, and others^5^, ^6^, ^7^. Patients with reflux commonly complain of hoarseness, chronic cough, globus pharyngis, odynophagia, dysphagia, and others, arising from the micro aspiration of acid into the larynx and pharynx, besides vagally mediated bronchi and laryngospasms^9^, ^10^.

The initial description of a possible association between GERD and laryngeal disorders was made in 1968 by Cherry & Margulies^11^ who reported three cases of patients with laryngeal granulomas associated with reflux esophagitis. Since then, studies have suggested otorhinolaryngological manifestations of this disorder, both in adults and in children alike, making it very clear that the diagnosis of the high manifestations of this disease ends up depending almost exclusively of a careful otorhinolaryngological examination, since it is only in 30% of the patients with this form of GERD that one can find esophageal or gastric disorders^12^. The importance and the impact of these atypical GERD manifestations are often times misunderstood; however, studies by Richter et al. in 1997, showed that 10% of the patients with chronic hoarseness may have GERD, this disease may be present in 30 to 89% of the patients with asthma, the acid reflux is the third cause of chronic cough and GERD is responsible for 50% of the chest pain events of non-cardiac origin^13^.

The laryngopharyngeal reflux is associated with the retrograde flow of the gastric content above the superior esophageal sphincter level, causing symptoms associated with the upper airway^14^ and it is one of the best known factors associated with the development of inflammatory disorders of the entire upper aero-digestive tract in children and in adults^15^, ^16^, ^17^, ^18^.

Monitoring through 24h pH probe is suggested as the most efficient method to investigate gastroesophageal reflux in children, and it is also possible to combine it with respiratory symptoms, having in mind that the patient is under care and monitoring for 24 hours, according to indications from the method^19^, ^20^. Nonetheless, despite the high incidence of reflux seen in the clinic, it is often times not diagnosed or suspected because of the large variability in symptoms.

Accurate information concerning GERD epidemiology is still under investigation, because of the difficulties caused by the variability in symptoms and the lack of a sensitive and specific diagnostic method. The present study assessed the prevalence of bronchopulmonary and otorhinolaryngological symptoms in children below 12 years of age submitted to 24 hour pH probe for diagnostic confirmation of the gastroesophageal reflux disease, with the goal of analyzing the association between these diseases, as well as to compare the frequency of these symptoms in the different age ranges and between children with positive and negative pH values.

## METHODS

This study was approved by the local Ethics in Research Committee, under protocol # 329/2007 and the parents/guardians of the children signed the Informed Consent Form.

This is a historical cohort study, based on the data recorded in outpatient ward medical charts from patients seen in the Pediatric Gastroenterology ward of the Pediatrics Department of a University Hospital. We assessed the charts from patients being seen for digestive complaints, with strong suspicion of gastroesophageal reflux, submitted to 24 hour pH probe (with the one or two channel device), located at 2cm and, when the second lead was present, at 5cm above the lower esophageal sphincter for diagnostic confirmation. We included children from both genders, at the age range between one and 12 years, all with clinical suspicion of GERD. We took off the study those patients with craniofacial malformations, cystic fibrosis, neurological disorders, immunoglobulin deficiencies, cow milk allergy and intolerance to acetyl-salicylic acid.

The children were broken down into three groups according to their ages: Group I, including children up to 24 months, Group II with those between 25 and 72 months and Group III, with those between 73 and 144 months of age (6 to 12 years).

From their charts we obtained the following data: gender, age at the time of the pH measuring and records of signs, symptoms or clinical hypotheses associated with the airway, grouped in bronchopulmonary disorders, such as chronic cough, recurrent bronchopneumonia and asthma; otologic symptoms such as recurrent otalgias, otitis before one year of age and recurrent otitis (records of 3 episodes within 3 months or 4 episodes within one year); nasosinusal symptoms (rhinosinusitis, pruritus and nasal obstruction) and upper airway infections, such as recurrent tonsillitis (6 episodes recorded within 1 year), recurrent pharyngitis and laryngitis. The presence of symptoms was described for the entire population studied and, for each age range. By the same token, we compared the presence of symptoms and disorders reported with the results from the pH measuring (positive or negative pH values) in each age range.

In order to compare the presence of clinical complaints and symptoms in the different age ranges, we used the Chi-Square test, using 5% as significance level (*p*<0.05).

In order to compare the presence of clinical complaints and symptoms among children with positive pH values with those with negative pH values, we also used the Chi-Square test, with a significance level of 5% (*p*<0.05).

## RESULTS

We assessed 143 charts from children who were submitted to 24 hour pH probe to investigate GERD between 1994 and 2007 at the Pediatric Gastroenterology service; however, only 65 matched the inclusion and exclusion criteria. Of the 65 children included, 37 were males; 36 (55%) children belonged to Group I, with age up to 2 years. Only five children aged below 6 years (7.7%) were referred to pH measuring in order to investigate GERD (Group III). The pH probe study was considered positive in 23 (35.4%) children, 12 (33.3%) from Group I, ten (41%) from Group II and only one (20%) from Group III. The distribution as to pH probe study positive results from each group is depicted on Table 1.

**Table 1:** Patient distribution according to the results from the pH probe study and age range.

	Group I	Group II	Group III
(+) pH probe test	12 (33,3%)	10 (41%)	1 (20%)
(-) pH probe test	24 (66,7%)	14 (59%)	4 (80%)
Total	36	24	5

Bronchopulmonary were the most prevalent symptoms in the children, found in 89.2%; there was a prevalence of nasosinusal symptoms (72.3%), ear symptoms (46.1%) and recurrent UAW infections (44.6%). When we compared the presence of each group of symptoms with the pH probe study, we did not find significant differences between the symptoms and the results from the pH probe study in the groups investigated.

The presence of bronchopulmonary symptoms was assessed in each age range, and we noticed that in Group I, of 36 children, 33 (91.6%) were symptomatic; in Group II, of 24 children, 23 (95.8%) had symptoms, but only two children (40%) from Group III, as per depicted on Table 2. When we associated the presence of respiratory symptoms with the results from the pH probe study, we did not find statistically significant differences between the positive and the negative pH probe studies.

**Table 2:** Symptom prevalence according to study group.s

	Bronchopulmonary symptoms	Otologic symptoms	Nasosinusal symptoms	Recurrent UAW infections
Group I n= 36	33 (91,6%)	16 (44,4%)	25 (69,4%)	13 (36,1%)
Group II n= 24	23 (95,8%)	12 (30%)	19 (79,2%)	15 (62,5%)
Group III n=5	2 (40%)	2 (40%)	3 (60%)	1 (20%)

The presence of otologic symptoms was assessed for each age range, and we noticed that in Group I, of 36 children, 16 (44.4%) had symptoms; in Group II, of 24 children, 12 (50.0%) had symptoms, and two children (40%) from Group III. When we associated otologic symptoms with the pH probe study results, we did not find statistically significant differences between positive and negative pH probe studies. When we investigated the presence of recurrent otitis associated with a positive pH probe study result, we noticed a significant difference (*p*<0.05), see Table 3.

**Table 3:** Recurrent otitis and their association with the pH probe study results.

	Recurrent Otitis	
	Symptoms present	No symptoms present
pH probe study (+)	11	13
pH probe study (-)	8	33
Odds ratio= 3.3	C.I (1.081-10.249) *p*< 0.05	

The presence of nasosinusal symptoms was assessed for each age range, and we noticed that for Group I, of 36 children, 25 (69.4%) had symptoms; in Group II, of 24 children, 19 (79.2%) had symptoms, and three children (60%) in Group III. When we studied the presence of nasosinusal symptoms with the results from the pH probe study, we did not find significant differences between the positive and the negative pH probe study results (table 4). Recurrent infections were assessed for each age range, and we noticed that in Group I, of 36 children, 13 (36.1%) had symptoms; in Group II, of 24 children, 15 (62.5%) were symptomatic, and two children (40%) from Group III.

**Table 4:** Presence of bronchopulmonary symptoms, otologic symptoms, nasosinusal symptoms, recurrent UAW infections in the children who were studied, and their association with the pH probe study.

n=65	Bronchopulmonary symptoms	Otologic symptoms	Nasosinusal symptoms	Recurrent UAW infections
pH (+) n (%)	21 (32,3%)	13 (20%)	18 (27,7%)	11 (16,9%)
pH (-) n (%)	37 (16,9%)	17 (26,1%)	29 (44,6%)	18 (27,7%)
Total n (%)	58 (89,2%)	30 (46,1%)	47 (72,3%)	29 (44,6%)

*p >0,05

## DISCUSSION

Initially, it was believed that in order to have high ENT manifestations upon GERD, it would be necessary to have, besides gastroenterological symptoms, also reflux esophagitis and hiatal hernia. Thus, it was possible to use diagnostic tests to confirm GERD, but it was not possible to confirm the presence of GERD in patients with ENT-related complaints only. Nonetheless, therapeutic tests were proven effective in these patients^17^, ^18^. Koufman^21^ finally showed that GERD can have two distinct clinical forms: the classic one, which courses with esophagitis and the second one, which may course with higher symptoms, being considered atypical, called laryngopharyngeal reflux. Such symptoms would be explained by the anatomy and physiology of the digestive and respiratory tracts.

In our study, all the children had strong clinical indications of having GERD; nonetheless, only 35% had positive pH probe study, despite the high frequency of gastroesophageal symptoms. In these children we could consider a diagnosis of atypical GERD. Another related factor which does not enable the diagnosis of GERD through the gold standard test - pH probe study, would be because it identifies only acid refluxes and the involvement of extra-esophageal structures could also happen due to basic reflux, as shown by Koufman^20^, ^21^.

When the children involvements were initially assessed according to age, we found a greater prevalence of individuals in Group I (36), although only 33.33% had a positive pH probe study. In Group III, we noticed a small group of children, with only five individuals. Nonetheless, at this age range an important remark to make, especially considering the extraesophageal complaints associated with the reflux, is if the incidence really reduces with age, or if GERD is no longer included as a differential diagnosis in the assessment of these patients.

The GERD ENT manifestations are atypical, for not initially remembering about the disease and because often times it does not go together with perceptible digestive symptoms. In the pediatric population, the diagnostic difficulties increases because of communication limitations associated with small children. The most frequent symptoms associating GERD with ENT manifestations in adults are dysphonia, hawking and the feeling of a foreign body in the pharynx^22^. These symptoms are evidently difficult to characterize in the pediatric population, and more common symptoms in this age range are retardation in growth and body weight, cough and recurrent respiratory or otologic infections. In the present study, it was possible to notice a greater prevalence of respiratory symptoms, present in 89.2% of the children, followed by digestive symptoms in 80% and nasosinusal in 72.3% who had clinical symptoms of GERD, and ear symptoms and infections were not so frequent, being present in 46.1% and 44.6% of the children, respectively, as previously depicted on Table 1.

In the pediatric population, rhinopharynx disorders happen more frequently, and it may cause recurrent adenoiditis and sinusitis and, because of Eustachian tube contiguity, recurrent otitis media or chronic secretory otitis media. In a study by Megale et al.^18^ 45 children with clinical diagnosis of GERD - proven by 24 hour pH probe study, from both genders, between 3 months and 12 years of age were assessed, in a retrospective study. Of these children, 51.11% had gastroesophageal symptoms, 40% recurrent pneumonias, 46.67% asthma, 64.44% had chronic cough, 68.88% nasal obstruction, 55.55% nasal secretion, 46.66% nasal pruritus, 35.56% recurrent acute otitis media and 24.44% recurrent tonsilitis^18^. And, as we can see in the same study, most of these symptoms had a significant cure rate, reaching almost 100%, since the gastroesophageal reflux was definitely treated, once again proving the close relationship between GERD and extraesophageal symptoms^18^. Among otologic symptoms, GERD has been associated with recurrent otitis^5^, ^6^, ^7^, and our study showed a statistically significant relationship between the children who had a positive 24h pH probe study and recurrent acute otitis media episodes.

Similar results were found in our study. Initially, of the 65 children referred to pH probe study, 24 had results matching those of GERD, corresponding to 36.9% of the initial sample. Of these, 58.3% had cough, 79.1% had recurrent bronchopneumonia; 16.7% had asthma; 8.3% apnea; 83.3% had digestive symptoms; 45.8% with rhinosinusitis; 25% with nasal pruritus; 58.3% had nasal obstruction; 41.6% had tonsillitis and 12.5% had pharyngitis.

Our study has limitations, especially because it is retrospectively based on reports and charts from children who were followed up by the pediatric gastroenterology department, without focus on otorhinolaryngological disorders; and therefore one cannot generalize the results for all the children followed in the otorhinolaryngology ward.

Typical reflux manifestations include regurgitation and vomit, which may happen, besides during the night as previously mentioned, also in the pre-prandial period. According to Karkos^9^, in a review published, 50% of normal children have regurgitation - with spontaneous resolution by 2 years of age. When not self-limited and really pathological, gastroesophageal reflux has manifestations which are very similar between adults and children^9^. In our study, digestive complaints were found in 80% of the children who were evaluated, being present in all the children older than six years. It may be that older children be referred only in the presence of digestive complaints, since the other groups of respiratory and infectious symptoms are less frequent than in the other age ranges and similar to nasosinusal and otologic symptoms of other age ranges.

The lack of statistical significance among extraesophageal symptoms and the Gastroesophageal Reflux Disease presented in the present study, does not rule out the importance and the relationship of these involvements for these patients. Statistically positive predictive data when comparing recurrent otitis and GERD - confirmed by pH probe study, already suggests to us that the patients may benefit from GERD treatment, besides the resolution of the gastroenterological complaints.

Patients with reflux frequently have ENT complaints, such as hoarseness, chronic cough, globus pharyngis, odynophagia, dysphagia, and others. These extraesophageal symptoms arise from the micro aspiration of acid into the larynx and pharynx, besides vagally mediated bronchi and laryngeal spasms. Nonetheless, these extraesophageal manifestations are often times underdiagnosed, or they are not associated with the gastroesophageal reflux because it is difficult to confirm them, or they may even be asymptomatic in some cases.

Our study stresses the frequency of otorhinolaryngological disorders in children whom, in the beginning, are seeing a doctor only because of their gastroenterological complaints, especially the younger ones. It is important to have a multidisciplinary approach, with professionals paying attention to the extraesophageal complaints, since the classical complaints may be harder for the children to express, and extraesophageal complaints are common to children of all ages.
